# News and Events of the Week

**Published:** 1910-07-16

**Authors:** 


### News and Events of the Week.

The King has been pleased to appoint Sir Alfred Downing Fripp, K.C.V.O., C.B., F.R.C.S., to be an honorary surgeon-in-ordinary to his Majesty.

The new pavilion of the National Sanatorium, Benenden, Kent, which has just been completed, will be opened by the Postmaster-General on September 12.

Dr. John M. Munro Kerr, Obstetric Physician to Glasgow Maternity Hospital and Gynæcological Surgeon to the Western Infirmary, formerly assistant to the Professor of Midwifery, Glasgow University, has been appointed to the Chair of Midwifery and Gynæcology in Anderson's College Medical School, Glasgow, in succession to the late Professor John Edgar.

Dr. Thomas Richards, of Temple House, Bath Row, Birmingham, medical officer and public vaccinator for the Edgbaston district of the King's Norton Union, who died on April 4, aged fifty-five, left estate of the gross value of £26,547, with net personalty £26,109. He left his collection of war medals to the colonel and officers for the time being of the 1st V.B. Warwickshire Regiment, whom failing, to the Birmingham Art Gallery.

The Provincial Sessional Meeting of the Royal Sanitary Institute will be held in the Ball Room of the Bath Saloons, Torquay, at 8 p.m., on July 22, when a discussion will take place on "The Water Supply, the Sewage Disposal, and the Climatic Conditions of Torquay." Tickets for admission of visitors may be had on application to Mr. H. A. Garrett, Assoc.M.Inst.C.E., Market Street Chambers, Torquay, who is kindly acting as the local hon. secretary of the meeting.

We have received a copy of the Great Eastern Railway Company's new handbook "East Coast Holidays," which can be obtained free on application to the Superintendent of the Line, Liverpool Street Station, London, E.C. This useful little guide gives particulars of most of the less-known districts in East Anglia, and of the country between the Cromer and Hunstanton coast and the Norfolk Broads. The illustrations have been made a special feature. Details of fares and tourists' tickets are given in an appendix.

Under the title "Medical Examination of Schools and Scholars" Messrs. P. S. King and Son, of Westminster, are about to issue a work which will be invaluable to school doctors, managers, teachers, educationists, and all interested in the welfare of the school child. The volume is edited by Dr. T. N. Kelynack, and Sir Lauder Brunton contributes the introduction. The book consists of thirty-two separate essays by experts in Great Britain, America, and the progressive countries of Europe.

Voting took place last week at the Royal College of Surgeons for the election of four Fellows into the council of the college. The President, Mr. Henry T. Butlin, announced the result of the poll to be as follows: Mr. Charles Barrett Lockwood, 532; Mr. Bilton Pollard, 514; Mr. John Bland-Sutton, 499; Mr. Charles Alfred Ballance, M.V.O., 484; Mr. James Ernest Lane, 383. The President declared Mr. Lockwood to be re-elected, and Mr. Bland-Sutton, Mr. Pollard, and Mr. Ballance to be elected. The poll was a heavy one, some 870 Fellows recording their votes.

The Tuberculosis Conference in Edinburgh has been sitting regularly, with Professor Osler (Oxford) in the chair. "The Avenues of Infection" has been discussed by Professor Sims Woodhead (Cambridge), who believed that in all probability tuberculosis had found its hold at some period of life in almost everyone. Professor Adams (Montreal) agreed that the majority of cases were caused by inhalation. The infection of the newborn was more frequent than was usually suspected. Infection in most cases was *post*-natal. Dealing with the Indians in the North-West of Canada, the speaker declared that practically every redskin child was infected before entering school. "Preventive Measures and Administrative Control" has been the subject treated by Dr. Hermann Biggs (New York). Other speakers have included Dr. Leslie Mackenzie (Edinburgh), Dr. Stafford (Dublin), Dr. Hope (Liverpool), and Dr. Scunfield (Sheffield).

A Dinner of the Brussels Medical Graduates' Association will be held at the Garden Club, Japan-British Exhibition, on Tuesday, July 19, at 7.45. Tickets 7s. 6d. (not including wine). The annual meeting and banquet of the Association will be held at the Brussels Exhibition on Saturday, August 6, at 7.30. Tickets 10 francs. Members are requested to let the hon. secretary know whether they can attend these functions. A seventeen-day excursion return ticket *viâ* Dover-Ostend to Brussels by any train, including saloon on steamer, costs £2 8s. 4d., and may be had from the agents (Messrs. Dean and Dawson, 84b Piccadilly. Headquarters in Brussels during the meeting, Wiltshires Hotel, Boulevarde de Waterloo, where a book will be kept for members in which to write their names and addresses as soon as they arrive. All foreign graduates will be welcome. A party will leave London on Friday, August 5, for Brussels. All applications to be made to Dr. Arthur Haydon, hon. secretary, B.M.G.A., 23 Henrietta Street, Cavendish Square, W.

The new Civil List pensions, details of which were issued last Saturday, include the following: Mr. Thomas Bryant, F.R.C.S. (£100), in recognition of his services towards the advancement of surgery, and in consideration of his age and reduced circumstances. Mrs. Mary Louisa Gamgee (£70), in consideration of the valuable contributions to physiological science of her husband, the late Professor Arthur Gamgee, and of her straitened circumstances. Mrs. Eleanor Jane Seeley (£70), in consideration of the valuable writings on geology and palæontology of her husband, the late Professor Harry Govier Seeley, and of her straitened circumstances. Miss Helena Stormont Murphy (£50), in consideration of the services rendered by her father, the late Professor Edward William Murphy, M.D., in furthering the use of chloroform, and of her straitened circumstances. Mr. James Sully, LL.D. (£95), in recognition of his services to psychology in addition to his existing pension (£105 in 1903). Mrs. Joanna Calder Fraser (£70), in consideration of the value of the investigations in anatomy and embryology of her husband, the late Professor Alexander Fraser, and of her inadequate means of support. Miss Julia Dobson (£15), in recognition of the important services rendered by her brother, the late Surgeon-Major George Edward Dobson, F.R.S., to zoological science, and in consideration of her straitened circumstances, in addition to her existing pensions (£25 in 1896 and £17 in 1904).

